# WNT links metabolism and cell cycle in postnatal cardiomyocytes

**DOI:** 10.20517/jca.2022.18

**Published:** 2022-04-18

**Authors:** Ivan Menendez-Montes, Hesham A. Sadek

**Affiliations:** 1 Department of Internal Medicine/Cardiology, University of Texas Southwestern Medical Center, Dallas, TX 75390, USA.; 2 Department of Molecular Biology, University of Texas Southwestern Medical Center, Dallas, TX 75390, USA.; 3 Department of Biophysics, University of Texas Southwestern Medical Center, Dallas, TX 75390, USA.; 4 Center for Regenerative Science and Medicine, University of Texas Southwestern Medical Center, Dallas, TX 75390, USA.

**Keywords:** WNT, cell cycle, metabolism, cardiomyocytes

Cardiovascular diseases are the leading cause of morbidity and mortality worldwide. Numerous cardiac pathologies result in myocardial damage or dysfunction, inevitably leading to heart failure. A fundamental roadblock in heart failure treatment is the inability of the adult human heart to repair itself after injury, which results from permanent cardiomyocyte cell cycle arrest shortly after birth. As such, mechanism(s) that regulate cardiomyocyte cell cycle progression have been the focus of intense research over the past two decades.

WNT/β-Catenin pathway is an evolutionarily conserved signaling pathway with essential roles in cell proliferation and differentiation. Canonical WNT signaling, mediated by the binding of WNT ligands to its membrane receptors, Frizzled and LRP5/6, and subsequent activation of downstream β-Catenin-dependent transcription has a pivotal role in the heart. During embryogenesis, WNT/β-Catenin signaling regulation is essential for the proper specification and proliferation of cardiac progenitor populations in the first and second heart fields^[1,2]^. Canonical WNT signaling has low activity in the adult heart^[3]^. However, this signaling pathway has also been shown to mediate cardiac pathology, such as cardiomyocyte hypertrophy and death^[4]^.

In this issue, Olcum *et al*.^[5]^ provide a deep analysis of the short-term transcriptional changes taking place upon WNT/β-Catenin signaling modulation in the adult mouse heart. By using cardiomyocyte-specific inducible gain (GOF) and loss (LOF) of function models of the canonical WNT pathway, the authors found that WNT/β-Catenin signaling governs the expression of cell cycle and oxidative phosphorylation-related genes in adult cardiomyocytes. Enhanced β-Catenin transcriptional activity in the GOF model resulted in increased expression of cell cycle re-entry and cytokinesis genes, while a decrease in the transcriptional activity of β-Catenin in the LOF model led to a modest increase of genes related to oxidative phosphorylation. Interestingly, none of the genetic models caused functional alterations in the heart, in terms of fibrosis, lipogenesis, cardiac function, cell size, or cardiomyocyte number.

Moreover, GOF animals did not show an increase in mitotic cardiomyocytes by means of pH3 immunostaining (although there was a trend towards increase, with considerable variability), a mitosis marker considered a gold standard for assessment of cardiomyocyte cell cycle entry, indicating that increased canonical WNT signaling may not be sufficient to induce cardiomyocyte proliferation under basal conditions. Additional studies, including S-phase and cytokinesis makers, would help clarify the effect of GOF on cardiomyocyte cell cycle. In addition, it would be important for future studies to determine the effect of GOF on cardiomyocyte cell cycle following ischemic injury.

Interestingly, the LOF model showed a modest increase in transcripts related to mitochondrial oxidative phosphorylation. These results, even if modest, would be interesting to pursue. For example, some functional experiments in the LOF model, such as mitochondrial activity, morphology, and substrate utilization, would be important to delineate the role of canonical WNT signaling in the mitochondrial biology of adult cardiomyocytes. Conceptually, a shift towards an oxidative phenotype is generally considered a facet of cardiomyocyte maturation. If true, although the picture is not complete at this stage, it would suggest that WNT signaling might regulate a cardiomyocyte maturation program involving metabolism and cell cycle regulation.

Previous studies in pathological cardiac conditions showed contradictory roles of WNT/β-Catenin signaling depending on the cell type studied. For example, inhibition of the canonical WNT pathway upon cardiac injury improves heart function and reduces fibrosis^[6,7]^. However, inhibition of the same pathway in epicardial cells and cardiac fibroblasts led to worsening of cardiac function and wound healing following myocardial infarction^[8]^. This highlights the importance of cell type-specific studies in regard to canonical WNT signaling in the adult heart. In this regard, Olcum *et al*.^[5]^ performed all the analyses using cell-specific genetic models, providing a cardiomyocyte-specific signature of transcriptional changes upon β-Catenin GOF and LOF in adult cardiac myocytes.

Previous studies have also demonstrated that β-Catenin is a component of the intercalated disk, and that it accumulates in that location during cardiac hypertrophy^[9]^ and aging^[10]^. Given the increased β-Catenin levels achieved in the GOF model in the current study, despite the fact that there does not seem to be any overt electrical or contractile consequences, it would be interesting to determine whether β-Catenin levels are also increased at the level of intercalated disks and whether some of the transcriptional changes or long-term effects are dependent on it.

WNT signaling is known to have a non-canonical pathway that acts independently of β-Catenin. However, there is increasing evidence that both WNT canonical and non-canonical WNT signaling interact and coordinately regulate several cellular processes^[11]^. In that regard, it is possible that some of the de-regulated pathways in the GOF and LOF models are due to alterations in the non-canonical branch of the pathway. Perhaps additional studies using double mutants of both β-Catenin and non-canonical WNT pathway members would help dissect the role of each signaling cascade in the adult cardiomyocytes.

In conclusion, Olcum *et al.*^[5]^ conducted a series of elegant studies that suggest a degree of regulation of cell cycle genes and cell cycle progression pathways by canonical WNT signaling in adult myocytes. As mentioned above, this does not appear to be sufficient for bona fide induction of cardiomyocyte mitosis in the adult heart under basal conditions; however, additional long-term and injury-related studies may be needed to further delineate the role of WNT signaling in myocardial regeneration following injury. From a mechanistic standpoint, it would be important to determine whether WNT signaling acts as a nodal point for the regulation of a cardiomyocyte maturation program involving metabolism and cell cycle.
